# Recent insights into the functions and mechanisms of antisense RNA: emerging applications in cancer therapy and precision medicine

**DOI:** 10.3389/fchem.2023.1335330

**Published:** 2024-01-10

**Authors:** Shahab Ur Rehman, Numan Ullah, Zhenbin Zhang, Yongkang Zhen, Aziz-Ud Din, Hengmi Cui, Mengzhi Wang

**Affiliations:** ^1^ College of Animals Science and Technology Yangzhou University, Yangzhou, China; ^2^ College of Animals Nutrition Yangzhou University, Yangzhou, China; ^3^ Department of Human Genetics, Hazara University Mansehra, Mansehra, Pakistan; ^4^ Institute of Epigenetics and Epigenomics Yangzhou University, College of Animal Nutrition Yangzhou University, Yangzhou, China

**Keywords:** AntisenseRNA, epigenetics regulation, cancer therapy, precision medicine, RNA biology

## Abstract

The antisense RNA molecule is a unique DNA transcript consisting of 19–23 nucleotides, characterized by its complementary nature to mRNA. These antisense RNAs play a crucial role in regulating gene expression at various stages, including replication, transcription, and translation. Additionally, artificial antisense RNAs have demonstrated their ability to effectively modulate gene expression in host cells. Consequently, there has been a substantial increase in research dedicated to investigating the roles of antisense RNAs. These molecules have been found to be influential in various cellular processes, such as X-chromosome inactivation and imprinted silencing in healthy cells. However, it is important to recognize that in cancer cells; aberrantly expressed antisense RNAs can trigger the epigenetic silencing of tumor suppressor genes. Moreover, the presence of deletion-induced aberrant antisense RNAs can lead to the development of diseases through epigenetic silencing. One area of drug development worth mentioning is antisense oligonucleotides (ASOs), and a prime example of an oncogenic trans-acting long noncoding RNA (lncRNA) is HOTAIR (HOX transcript antisense RNA). NATs (noncoding antisense transcripts) are dysregulated in many cancers, and researchers are just beginning to unravel their roles as crucial regulators of cancer’s hallmarks, as well as their potential for cancer therapy. In this review, we summarize the emerging roles and mechanisms of antisense RNA and explore their application in cancer therapy.

## 1 Introduction

**FIGURE 1:**
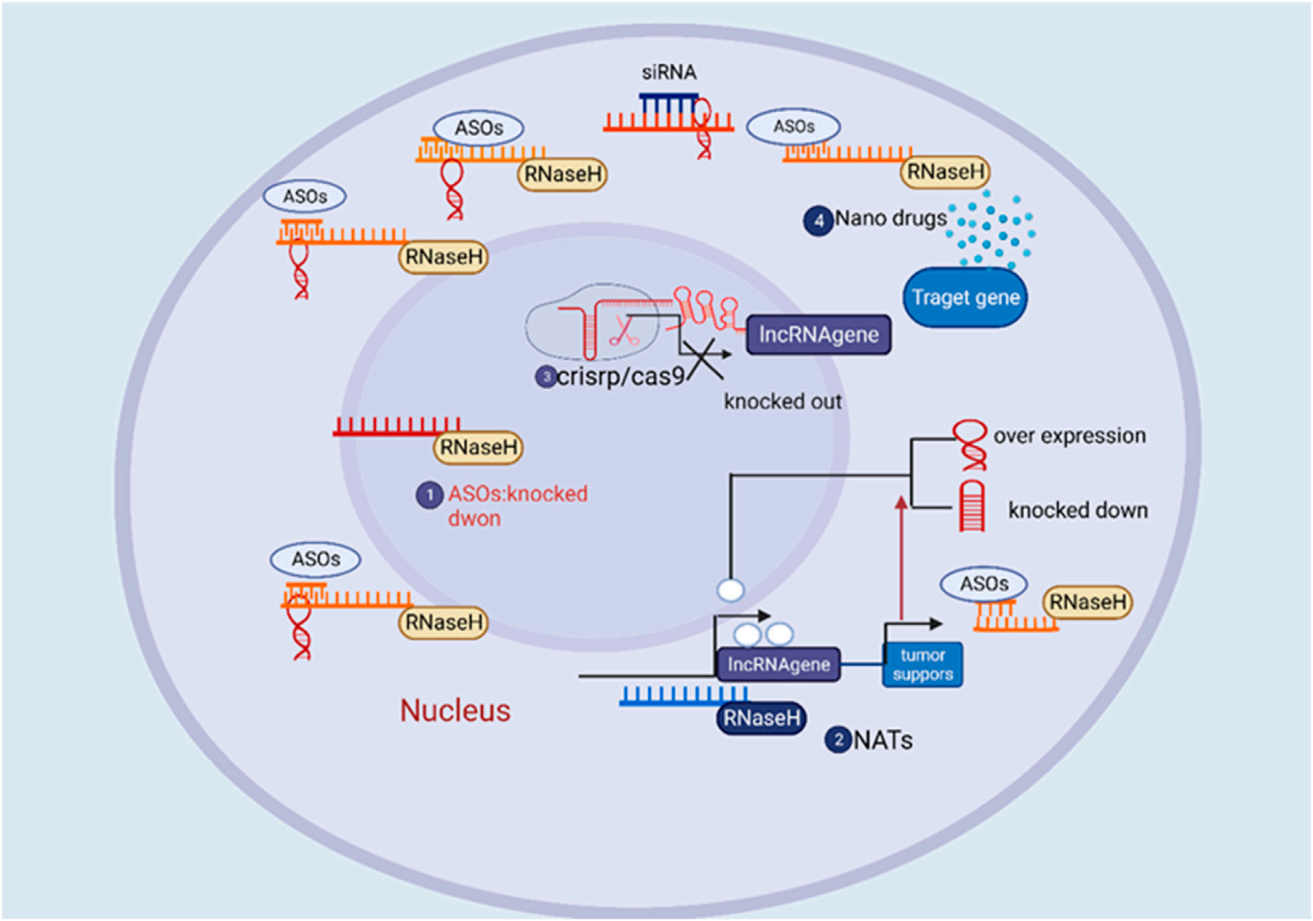
The overview of the therapeutic approach of directing therapeutic agents towards long non-coding RNA (lncRNA) for the treatment of cancer.

Antisense transcripts, which were previously regarded as mere transcriptional noise, are currently recognized as significant regulators of gene expression (Kumar and Carmichael, 1998). It is now widely understood that antisense RNAs (asRNAs) are abundant across all biological kingdoms. An estimated 50% of messenger RNAs (mRNAs) in human cells are accompanied by corresponding antisense transcripts (Brantl, 2002). Non-coding RNAs, specifically asRNAs, play a unique and pioneering role in the intricate network governing gene expression (Fang and Fullwood, 2016). asRNAs significantly influence the cellular function of animals and contribute to the pathological processes that underlie various human illnesses. Specifically, in cancer biology, asRNAs play a crucial role in regulating cellular development, cell cycle progression, cellular differentiation, as well as the processes of invasion and metastasis (Werner, 2013). asRNAs play a crucial role in the initiation and advancement of diverse cancer types, either by promoting carcinogenesis as “Onco-asRNAs” or by inhibiting carcinogenesis as “Ts-asRNAs (Yu et al., 2008)”. The crucial involvement of asRNAs in tumor development becomes apparent as their expression is disrupted and they exert influence over protein-coding genes. In malignant cells and tissues, the prevailing trend is an increase in the expression of oncogenic asRNAs, whereas tumor suppressor asRNAs tend to be frequently downregulated (Iacobucci et al., 2010). The study of noncoding RNAs (ncRNAs) has gained considerable momentum in the past decade due to the identification of various influential ncRNA categories, such as small interfering RNA (siRNA), microRNA (miRNA), long noncoding RNA (lncRNA), and PIWI-interacting RNA (piRNA) (Richard Boland, 2017). A study administered pelacarsen subcutaneously to 286 individuals with established cardiovascular disease (CVD) and elevated lipoprotein (a) [Lp(a)] levels. It was observed that Lp(a) levels decreased in a dose-dependent manner, ranging from 35% to 80%, with a favorable safety profile. Pelacarsen, an antisense oligonucleotide (ASO) medication candidate for CVD, has demonstrated promise in phase 2 trials and is currently undergoing evaluation in phase 3 studiesThe majority of long noncoding RNAs (lncRNAs) exhibit an antisense orientation, but it is also important to recognize the existence of intronic lncRNAs, intergenic lncRNAs, and bidirectional (or divergent) lncRNAs (Latgé et al., 2018). Antisense long non-coding RNAs (lncRNAs) are synthesized from the complementary strand of genes that possess either protein-coding or non-coding functions (Bhan et al., 2017). Similar to ncRNAs, antisense lncRNAs are categorized based on their proximity to protein-coding genes, yet they lack the ability to encode proteins. The production levels of antisense lncRNAs vary across different cell types, exerting control over the activity of particular genes and specific signaling pathways (Zhao et al., 2020). Interestingly, either these entities could serve a role in the regulation of genes, in cis or trans. Trans-acting antisense long non-coding RNAs (lncRNAs) exert their impact on the expression of other genes through partial sequence complementarity. In contrast, cis-acting antisense lncRNAs modulate the expression of the genes from which they originate by interacting with the promoter region in a manner that involves absolute sequence complementarity (Pelechano and Steinmetz, 2013). Similar to ncRNAs, they are classified based on their proximity to protein-coding genes but cannot be translated into proteins. Different cell types have varying production levels of antisense lncRNAs, which control the activity of certain genes and specific signaling pathways (Zhao et al., 2020). The available research data derived from published studies unequivocally establish that specific asRNAs possess the capability to initiate carcinogenesis or aid in the progression of cancer (Potaczek et al., 2016). On the other hand, certain asRNAs have protective properties and can even demonstrate therapeutic efficacy in malignant diseases by suppressing the oncogenic potential within cells (Sullenger and Nair, 2016). Co currently with the swift acquisition of information regarding antisense RNAs (asRNAs), there is a quick emergence of technologies that use these molecules as therapeutic targets or agents (Naidu et al., 2015). These advancements hold significant potential for future applications in the treatment of human malignancies (Figure 1).

This review article presents a comprehensive compilation of current knowledge on antisense RNAs, focusing specifically on their synthesis, functioning, and regulatory effects on target gene expression. Additionally, we examine the therapeutic potential of asRNAs in cancer treatment. Moreover, we explore the crucial role that antisense RNAs play in governing gene expression and their potential therapeutic applications in combating viral infections and cancer.

## 2 Research history of antisense RNA

**FIGURE 2:**
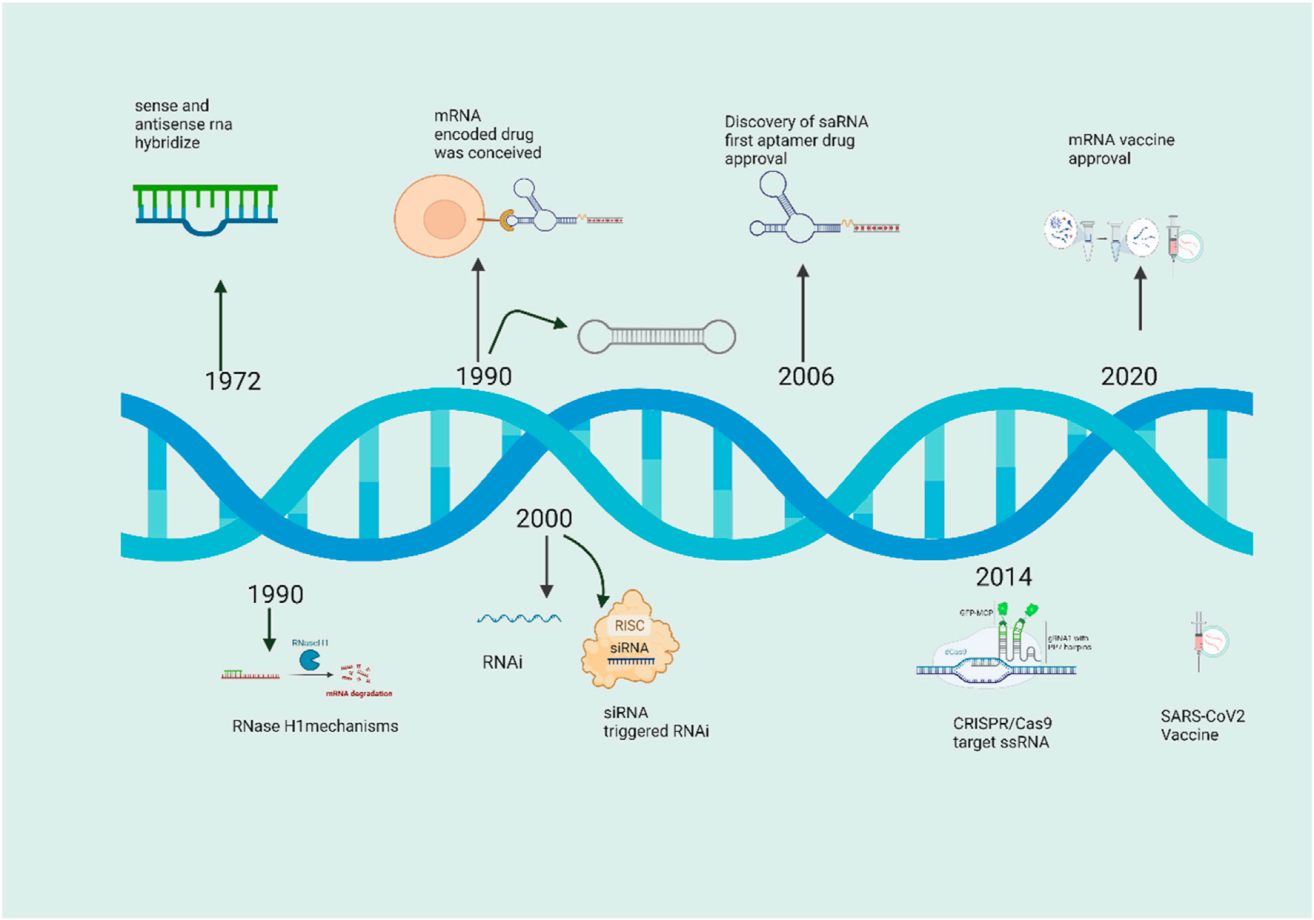
Brief history of RNAs and antisense RNA in from 1972 to 2022.

Although the history of scientific exploration spans a vast and intricate landscape, our focus centers on the nucleic acid-based gene silencing/modification field, delving into significant research, facts, and findings. Acknowledging the extensive literature on antisense RNAs, we recognize the necessity of a selective approach. The practical application of antisense oligonucleotides (ASOs) traces its roots back to 1967 in Novosibirsk (Russia) when Grineva pioneered the concept (Georg et al., 2009). Her groundbreaking proposal involved attaching active chemical groups to oligonucleotides, guiding these groups to specific nucleic acid fragments complementary to the oligonucleotide. This directed chemical reaction, termed the “method of complementary-addressed modification,” facilitated modifications in targeted regions of the nucleic acid near the formed duplex structure. In 1977, Grineva and her colleagues displayed the effectiveness of the antisense approach by modifying valine tRNA, demonstrating alkylation with a reagent bound to the corresponding oligonucleotide at specific points along the valine tRNA. Nobel laureate Gobind Khorana, in 1972, devised a strategy for synthesizing a DNA duplex with a sequence mirroring the major yeast alanine transfer RNA using overlapping DNA oligonucleotides (Figure 2).

The initial documentation of the *in vivo* repression function of natural antisense RNAs occurred in the early 1988, specifically in plasmid CoIEI of bacteria. Following the CoIEI discovery, various systems were characterized, revealing instances where antisense RNA plays a role in inhibiting gene expression to regulate processes such as plasmid replication, transposition, and conjugation in prokaryotes. In the realm of eukaryotic cells, Izant and Weintraub were pioneers in demonstrating the functionality of antisense gene constructs. They accomplished this by modifying a vector, excising the herpes simplex virus (HSV) thymidine kinase gene (TK)*,* and subsequently reinserting it into the vector in a reverse orientation. When co-injecting the modified and unmodified vectors with wild-type *TK* into TK mouse L cells, they observed a fourfold drop in *TK* activity (Izant and Weintraub, 1984). One year later, these researchers introduced antisense plasmid constructs into eukaryotic cells through methods such as transfection or microinjection. They conducted both transient and stable transformation assays, revealing that antisense transcripts complementary to as few as 52 nucleotides of the 5′ untranslated target gene mRNA could effectively suppress gene activity. This provided additional evidence that the mechanism of suppression by antisense RNA is conserved in eukaryotic cells. In the same timeframe, two separate research groups independently displayed the phenomenon of RNA antisense repression in higher organisms. One group utilized a SP6 vector in an *in vitro* transcription system to generate Kr antisense RNA, which was subsequently injected into wild-type *Drosophila* embryos. This resulted in the inactivation of Kr, leading to the development of *Drosophila* embryos with phenotypes resembling those of Kr mutant embryos (Rosenberg et al., 1985). Two independent research teams working with *Xenopus* have identified endogenous antisense RNA for basic Fibroblast Growth Factor (bFGF), suggesting a potential role in modulating the stability of bFGF mRNA (Kimelman and Kirschner, 1989). An additional observation revealed that nuclear antisense RNA induces adenosine deamination, leading to nuclear transcript retention, and subsequently contributing to transcript degradation (Kumar and Carmichael, 1997). In 2003, a study presented the inaugural evidence indicating that a deletion in LUC7L gives rise to juxtaposition with a neighboring HBA2 gene and the generation of antisense RNA. This event culminates in the epigenetic silencing of HBA2 in a patient affected by α-thalassemia (Tufarelli et al., 2003). A multitude of natural antisense RNAs exhibiting repressive functions have been discerned across diverse species. Ongoing exploration of genome-wide antisense transcription is presently underway in various animal and plant species, facilitated by advancements in laboratory tools. Noteworthy instances of such species encompass humans, mice, rice, and *Arabidopsis thaliana* (Chen et al., 2004; Wang X. J. et al., 2005). The inaugural systematic identification of genome-wide sense-antisense transcripts through molecular biological methodologies was executed in the year 2004 (Wang X. J. et al., 2005). Devised an experimental methodology enabling the genome-wide identification of endogenous mRNA featuring extended complementary sequences to other transcripts in both human normal mammary epithelial and breast cancer cells (Røsok and Sioud, 2004; He et al., 2008; Yu et al., 2008; Raal et al., 2010; Geary et al., 2015) In a parallel endeavor, He et al. introduced a technique named Asymmetric Strand-Specific Analysis of Gene Expression (ASSAGE), which facilitated the comprehensive identification of sense and antisense transcripts from expressed genes across five distinct human cell types. While conventional understanding posited that gene regulation by antisense RNA primarily occurs post-transcriptionally, functioning solely as a repressor of mRNA translation, a recent investigation challenged this notion. This study demonstrated that antisense RNA might exert silencing effects on its sense gene at the transcriptional level. This impact involves influencing A study administered pelacarsen subcutaneously to 286 individuals with established cardiovascular disease (CVD) and elevated lipoprotein (a) [Lp(a)] levels. It was observed that Lp(a) levels decreased in a dose-dependent manner, ranging from 35% to 80%, with a favorable safety profile. Pelacarsen, an antisense oligonucleotide (ASO) medication candidate for CVD, has demonstrated promise in phase 2 trials and is currently undergoing evaluation in phase 3 studies. However, the European Medicines Agency ultimately decided against approving Mipomersen despite these positive findings because of safety concerns related to liver damage. Although it gained FDA approval in 2013, its usage has been limited by safety concerns and the availability of alternative therapies (Sochol et al., 2019). Inotersen, like Mipomersen, is a GAPmer-structured, 2′-O-MOE-modified ASO from the second generation. Using it to treat transthyretin amyloidosis has been given the go-light as of 2018 (Keam, 2018).

The spliceosome is a complex molecular machinery responsible for removing introns from transcribed pre-mRNA, enabling the connection of exons to generate mature mRNA. ASOs can regulate spliceosome activity by binding to specific sequences in pre-mRNA, leading to the exclusion or inclusion of exons, thereby modifying the resulting mRNA. Dystrophin is a vital protein for maintaining muscle fiber integrity. Mutations in the dystrophin gene result in the absence or dysfunction of dystrophin, causing Duchenne muscular dystrophy (DMD). ASO-mediated exon skipping aims to restore the production of partially functional dystrophin proteins to alleviate DMD symptoms. Eteplirsen is a splice-modulating oligonucleotide used in the therapeutic management of individuals with DMD. In DMD, exon skipping targets the restoration of the reading frame of the disrupted dystrophin gene, leading to the synthesis of a shorter yet partially functional dystrophin protein. Eteplirsen is effective for approximately 13%–14% of DMD patients who carry specific mutations. To address additional splicing defects in DMD, approved drugs such as golodirsen, viltolarsen, and casimersen are available. These pharmaceutical agents facilitate the skipping of exon 53 or 45, thereby enhancing dystrophin protein production. All these pharmaceutical substances, including eteplirsen, belong to the third-generation antisense oligonucleotide (ASO) medications, distinguished by their advanced chemical modifications called phosphorodiamidate morpholino oligomers (PMOs) (Nakano et al., 2010). The urgency of patients’ clinical situations has led to the development of patient-customized oligonucleotide treatments. Milasen is an example, being the first patient-customized ASO used to treat neuronal ceroid lipofuscinosis 7, a fatal neurodegenerative disease. Milasen’s therapy showed acceptable side effects (Heo, 2020; Kim et al., 2020). It is worth noting, that the emergence of patient-customized treatments signifies a noteworthy advancement in the field of antisense RNA therapeutics, presenting a novel approach to address diseases that exhibit limited response to conventional drug regimens. Antisense RNA therapeutics operate through targeted manipulation of the expression and activity of specific molecules, thereby offering promising prospects for personalized medicines catering to the needs of individuals affected by rare diseases.

Ongoing clinical trials are being conducted to investigate the therapeutic potential of ribozymes in the treatment of solid tumors, HIV, and several other disorders. One such ribozyme, RPI.4610 (Angiozyme), targets the vascular endothelial growth factor receptor one (VEGFR1) mRNA to inhibit angiogenesis (Morrow et al., 2012). However, its poor efficacy has hindered further clinical development (Mitsuyasu et al., 2009; Karuppiah et al., 2021). This suggests cell-mediated gene delivery could be a safe therapeutic approach for HIV patients and potentially a conventional HIV treatment (Mitsuyasu et al., 2009). While the earliest clinical studies with ribozymes showed promising outcomes, further research is required to ascertain their stability, *in vivo* functionality, targeted delivery to specific tissues, and sustained expression over an extended period of time. Addressing these aspects will be crucial for successfully implementing ribozymes as therapeutics. Transthyretin is a plasma protein produced in the liver that, along with the retinol-binding protein complex, is responsible for transporting the thyroid hormone thyroxin throughout the body. Amyloid deposits in various organs result from single-nucleotide polymorphisms in the transthyretin gene, including coding and non-coding regions (Sikora et al., 2015). Patients given weekly subcutaneous injections of Inotersen showed reduced levels of circulating transthyretin, slowed disease progression, and enhanced quality of life in a randomized, double-blind, placebo-controlled phase 3 clinical study (Benson et al., 2018). Oligonucleotides have become a powerful tool for treating genetic diseases and cancer by altering protein synthesis. Methods for transporting oligonucleotide drugs include bio conjugation to escort moieties, chemical modification of nucleic acids, and development of nanoparticle carriers. Many medications have been brought to clinical approval thanks to these technical advancements, but the problem of efficient and focused distribution persists.

## 3 Characterization of antisense RNA

Lehner et al. (2002); Yelin et al. (2003); Collani and Barcaccia (2012); Loganathan and Doss C (2023) A study administered pelacarsen subcutaneously to 286 individuals with established cardiovascular disease (CVD) and elevated lipoprotein (a) [Lp(a)] levels. It was observed that Lp(a) levels decreased in a dose-dependent manner, ranging from 35% to 80%, with a favorable safety profile. Pelacarsen, an antisense oligonucleotide (ASO) medication candidate for CVD, has demonstrated promise in phase 2 trials and is currently undergoing evaluation in phase 3 studies In the context of eukaryotes, viruses, and notably the human immunodeficiency virus type one (*HIV-*1), have garnered considerable attention from scientists. This study specifically investigated the *HIV*-1, revealing the identification of functional antisense RNAs transcribed from the viral genome (Berro et al., 2007). The findings indicate that these antisense transcripts possess the capability to affect viral replication through the modulation of gene expression. The exploration of antisense RNAs in HIV-1 infection holds promise in elucidating the complex mechanisms underlying viral gene regulation and may pave the way for the development of innovative antiviral therapies (Streib et al., 2007). These antisense RNAs have been found to modulate the expression and replication of HIV-1 genes. Thus providing evidence that antisense RNA-mediated silencing of viral genes might be an effective antiviral therapy target (Berro et al., 2007).

Piero Carninci *et al,* conducted a study to better understand the breadth and possible functional roles of these non-coding RNA molecules, the researchers set out to discover and describe all antisense transcripts in the mammalian transcriptome (Katayama et al., 2005b). In the study, publicly available databases and freshly developed RNA sequencing (RNA-seq) data were used to collect transcriptome data from diverse mammalian species, including humans and mice. The team created methods to compare sequencing reads to identified sense transcripts or mRNAs to detect antisense transcripts (Lindberg and Lundeberg, 2010). Researchers annotated genomic locations, lengths, and other features of antisense transcripts. They compared antisense and sense transcript expression levels (Katayama et al., 2005a). Functional enrichment analysis revealed the antisense transcripts’ putative roles. Associating antisense transcripts with Gene Ontology (GO) keywords, biochemical pathways, and biological processes helped reveal their possible functions in cellular regulation (Katayama et al., 2005b). The collaborative investigation revealed the frequency and characteristics of antisense transcripts in the mammalian transcriptome (Deveson et al., 2017). This investigation significantly advanced our understanding of the intricate regulatory mechanisms governing gene expression in mammals. Specifically, it illuminated the prevalence and functional implications of antisense transcription. Researchers in this study focused on the impact of antisense long noncoding RNAs (lncRNAs) on morphine tolerance in mice. Notably, certain antisense lncRNAs exhibited elevated expression levels following long-term morphine therapy, and their influence was demonstrated in modulating the expression of genes encoding opioid receptors, thereby affecting morphine sensitivity and tolerance. Lu Z. et al. (2020).

Antisense RNA characterization is a new and quickly developing area of study. A multitude of antisense transcripts with diverse functions has been uncovered, providing insights into their regulatory roles in gene expression. Potential applications in biotechnology and medicine hinge on understanding the processes of antisense RNA-mediated gene control, which is still a topic of ongoing research. Undoubtedly, further studies in this area will help us better grasp how genes and cells function together.

## 4 Function of antisense RNA

The transcript or the transcription process can mediate an antisense transcript’s function. In addition, the functional effects of antisense expression can be classified as either cis (affecting only the alleles on the strand of DNA from which they are produced, typically locally) or trans (affecting alleles on different strands of DNA) in nature (Berretta et al., 2008). When antisense effects are observed in trans, the commonly held assumption is that the transcribed RNA molecules are accountable for these effects. Conversely, antisense effects in cis are generally attributed to the process of antisense transcription itself. However, both interpretations are incorrect (Wery et al., 2011). Interactions mediate trans effects between antisense transcription regions and other loci via the three-dimensional organization of chromatin, and cis effects are mediated by the persistence of antisense transcripts at the sites of their synthesis (via stalled polymerases, R-loops or triple helices, for example,) (Camblong et al., 2009). The fact that both antisense and sense transcripts are produced from the same area implies that antisense transcripts function more frequently in cis than other ncRNAs that usually operate in transcription rather than in cis (Camblong et al., 2007; Rinn et al., 2007). Modulating a gene’s expression and then analyzing the resulting changes in phenotype is a tried-and-true method for identifying its role (Woo et al., 2017). However, their chromosomal location makes antisense transcripts notoriously difficult to manipulate without altering sense expression. Antisense RNA (asRNA) is essential in controlling gene expression in many different types of organisms. It is an antisense molecule to the sense RNA, also known as messenger RNA (mRNA), generated from the DNA strand encoding the protein (Ma et al., 2021). Different regulatory effects, which affect gene expression at varying levels, might result from antisense RNA interacting with its target mRNA (Statello et al., 2021).

### 4.1 Epigenetics regulation

Epigenetics is commonly described as alterations in gene expression that are passed down without alterations to the DNA sequence. Recent studies indicate that the impact of antisense long noncoding RNAs (lncRNAs) on gene expression involves influencing epigenetic modifications, including DNA methylation and histone modifications. DNA methylation, a crucial epigenetic mechanism regulating gene expression, plays a significant role in cancer development, highlighting the importance of alterations in DNA methylation patterns (Meissner et al., 2008).

A study administered pelacarsen subcutaneously to 286 individuals with established cardiovascular disease (CVD) and elevated lipoprotein (a) [Lp(a)] levels. It was observed that Lp(a) levels decreased in a dose-dependent manner, ranging from 35% to 80%, with a favorable safety profile. Pelacarsen, an antisense oligonucleotide (ASO) medication candidate for CVD, has demonstrated promise in phase 2 trials and is currently undergoing evaluation in phase 3 studies. Similarly, *AGAP2*-AS1 induces histone acetylation in the MYD88 promoter region, which stimulates cell proliferation and suppresses apoptosis in breast cancer (BC). Furthermore, certain antisense long noncoding RNAs (lncRNAs) have been implicated in additional histone modifications. Specifically, in gastric cancer (GC), the heightened expression *of FOXD2*-AS1 facilitates carcinogenesis by orchestrating the epigenetic silencing of *EPHB*3. This is achieved through the recruitment of *EZH*2 and *LSD1*, resulting in *H3K27* methylation and *H3K4* demethylation, respectively (Xu T. P. et al., 2018).

### 4.2 Antisense transcriptional activation

**FIGURE 3:**
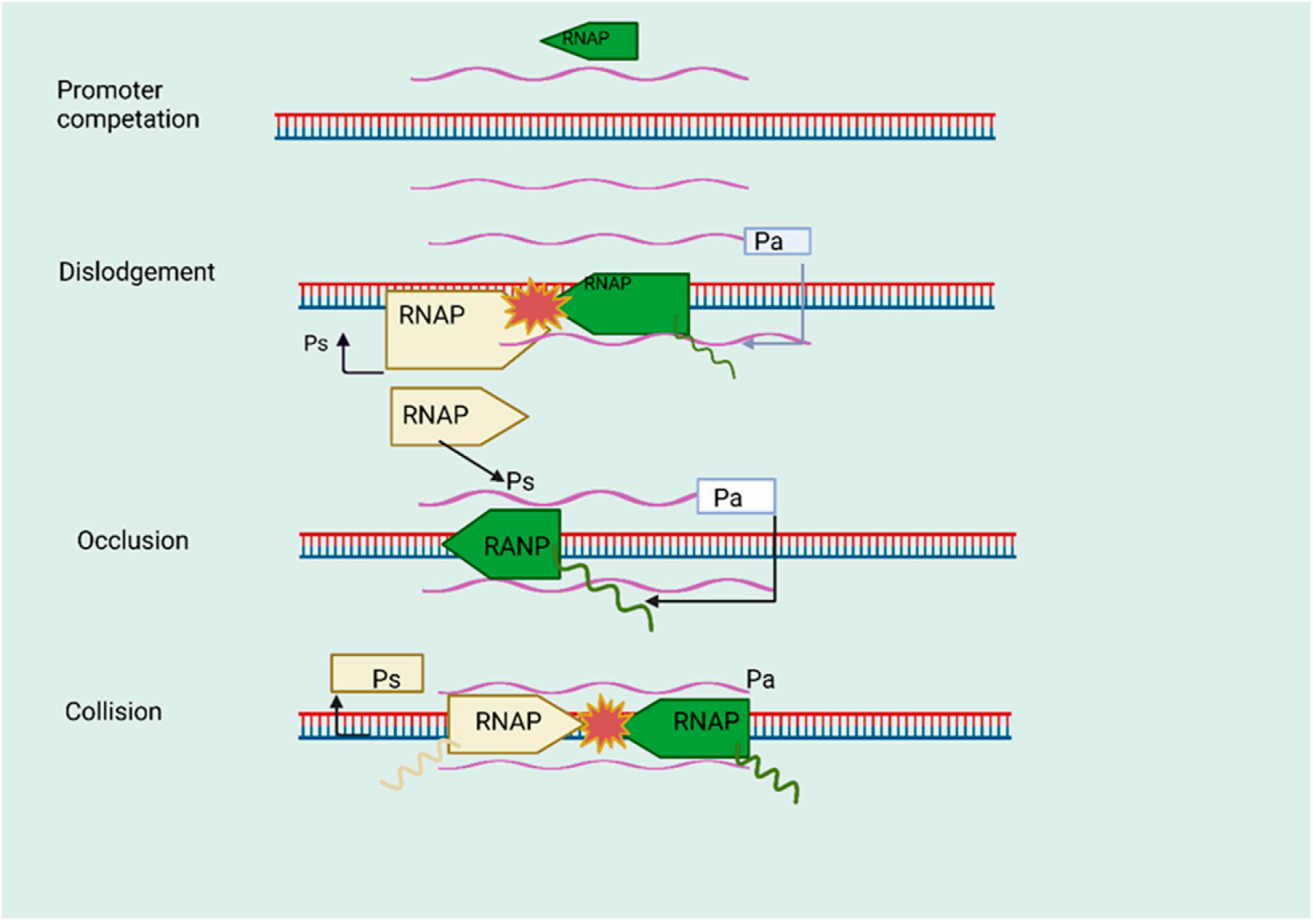
The phenomenon of transcriptional interference facilitated by natural antisense transcripts (NATs). During the initiation phase, namely, in the initial stage denoted as promoter competition, a competitive interaction occurs between the promoters of sense and antisense genes in order to secure the RNA polymerase (RNAP) complex. The introduction of the antisense gene RNAP complex results in the displacement of the RNAP complex associated with the promoter of the sense gene. During the process of antisense transcript elongation, the complex formed by the antisense RNA polymerase (RNAP) obstructs the promoter region of the sense gene. During the elongation stage of transcription. The phenomenon of collision arises when RNA polymerase (RNAP) complexes come into contact inside the region where sense and antisense genes overlap, thereby impeding the ongoing process of transcription.

Positive regulation of gene expression by antisense RNA occurs via increased transcription. In this case, the antisense RNA binds to regulatory elements such as promoters and enhancers of the target gene (Gil and Ulitsky, 2020). This binding might stabilize transcription factors or chromatin-modifying complexes to assist transcription initiation and elongation better, resulting in an open chromatin shape (Gil and Ulitsky, 2020). Shearwin et al. have developed multiple theoretical models to offer a deeper understanding of the fundamental mechanisms involved in antisense-mediated transcriptional interference (Zhao et al., 2020). These models shed light on the potential occurrence of such interference not only during transcription start but also throughout the process of transcription elongation. (A) Promoter competition occurs when the presence of an antisense promoter limits the occupancy of RNA polymerase (RNAP) at the sense promoter, leading to the downregulation of both transcripts. (B) Dislodgement refers to the sensitivity of the sense promoter to RNAP binding, which is a slow process, resulting in displacement by the antisense RNAP complex. (C) Occlusion describes the situation where the RNAP complex obstructs the sense promoter, preventing its proper functioning. The first three of these models could be associated with the initiation phase of sense gene transcription, while the last two may play a role in the elongation phase of sense gene transcription (Shearwin et al., 2005). Figure 3, however, is unclear that such simplistic models can account for all aspects of transcriptional interference. The study of transcriptional interference in higher eukaryotes is intricate due to factors like the diversity of transcription initiation complexes and the intricacies of chromatin structure. Several examples of antisense-mediated transcription activation in genes involved in development, stress responses, and immunological control (Rahman et al., 2017). The investigation focused on the role of antisense RNA in enhancing LRP1 gene transcription. An antisense transcript from the LRP1 locus was shown to interact with HMGB2, a protein found in chromatin (Shinohara et al., 2017). Because of this interaction, RNA polymerase II and other components of the transcriptional machinery were brought to the promoter region of the sense LRP1 gene, leading to the activation of transcription. The research showed that antisense RNA may positively regulate gene expression by altering chromatin structure (Yamanaka et al., 2015). The potential of antisense RNA in gene silencing through transcription was examined in *Escherichia coli*. It was found that transcriptional repression occurred when antisense RNA molecules were targeted at the promoter region of specific genes. The target genes were silenced by transcriptional silencing due to this method (Overlöper et al., 2014). Although this work focused on transcriptional repression, the ability of antisense RNA to modulate gene expression in either a positive or negative direction was highlighted (Georg et al., 2020). Alternative splicing (AS) is frequently linked to the process of transcription, and long non-coding RNAs (lncRNAs) have the potential to modulate AS by means of transcriptional control (Olivero et al., 2020). Lung cancer growth is inhibited by the lncRNA Pvt1b, which reduces the transcriptional activity and overall level of c-Myc (Olivero et al., 2019). The well-documented phenomenon involves the oncogenic transcription factor c-Myc causing the deregulation of pyruvate kinase mRNA splicing. This occurs via the transcriptional activation of hnRNP proteins (David et al., 2010). (HCC) also exhibits carcinogenic activity due to an increase in the lncRNA MALAT1 (Metastasis-associated lung adenocarcinoma transcript 1) (Malakar et al., 2017). Mechanism analysis indicated that MALAT1 targets the Wnt pathway/c-Myc axis to stimulate the production of the oncogenic splicing factor SRSF1 (Malakar et al., 2017). Furthermore, investigating the function of antisense transcriptional activation in development, tissue-specific expression, and numerous illnesses would be of interest. Understanding how this regulatory system contributes to normal cellular activities and abnormal circumstances might have major implications for therapeutic approaches.

### 4.3 Alternative splicing regulation

Alternative splicing is a crucial mechanism that allows a single gene to produce multiple protein isoforms, thereby expanding proteomic diversity. It plays a significant role in various biological processes, including cell differentiation, and its dysregulation has been implicated in disease states. Eukaryotic gene expression relies heavily on alternative splicing, allowing various mRNA isoforms to be generated from a single gene by selectively incorporating or deleting exons (Wright et al., 2022). This mechanism allows the production of many proteins from a single gene, each serving a unique purpose. Development, tissue-specific expression, and the response to environmental signals rely heavily on the closely regulated process of alternative splicing (Nembaware et al., 2008). Antisense RNA continues to be a subject of active research due to the limited understanding of its role in splicing. The antisense strand of an RNA molecule is a sequence of nucleotides complementary to a gene’s sense strand. Antisense RNA may affect splicing because of RNA-RNA interactions, in which it may build double-stranded RNA structures with the sense RNA (Santos et al., 2022). By forming complementary base pairs with pre-mRNA, antisense RNA molecules may interfere with correctly identifying splice sites and splicing regulatory elements. Exon inclusion or exclusion is regulated by the degree to which this interaction promotes or inhibits the use of certain splice sites (Santos et al., 2022). Traditionally, NATs interact with certain splicing factors to regulate alternative splicing (Romero-Barrios et al., 2018). The role of the antisense non-coding RNA EGOT, which is transcribed from the sense ITPR1, involves the stimulation of autophagy and enhancement of the susceptibility of breast cancer cells to paclitaxel, an anti-cancer agent that impedes mitosis and diminishes the proliferation of cancerous cells (Xu et al., 2019). ITPR1 overexpression can be achieved because EGOT can muddle to the ITPR1 pre-mRNA, which recruits the splicing factor hnRNPH1 through motif-specific recruitment. As an oncogene, EGOT functions this way (Xu et al., 2019). Additionally, NAT may directly disturb splicing variant equilibrium by creating an RNA-RNA duplex with its sense RNA to hide splice sites. ZEB2-AS1, ZEB2’s NAT, is one such well-known instance (Pelechano and Steinmetz, 2013). ZEB2, a transcription factor that serves as a biomarker for the process of endothelial-mesenchymal transition (EMT), exerts a significant influence on the intricacies of cancer. Since ZEB2-AS1 overlaps with the intron of ZEB2’s 5′UTR (untranslated region), which includes an internal ribosome entry site (IRES) essential for ZEB2 translation, an RNA-RNA duplex is formed to shield the IRES from processing, which improves ZEB2’s translation efficiency (Beltran et al., 2008). This is likely what motivates EMTs in some forms of malignancy. Antisense RNA-mediated splicing control is of great interest because of its potential impact on human illness. New treatment approaches may be possible if we learn how this mechanism’s dysregulation leads to various clinical diseases.

### 4.4 Post-transcriptional

Post-transcriptional gene regulation involves the control of gene expression after the transcription of RNA from DNA. This process includes RNA splicing, RNA editing, and the regulation of mRNA stability and translation. It is a critical step in the fine-tuning of gene expression and the generation of proteomic diversity. Many antisense lncRNAs function as ceRNAs at the post-transcriptional stage, controlling the development and progression of cancer. CeRNAs refer to competitive endogenous RNAs, which are messenger RNAs that are targeted by microRNAs (miRNAs). These CeRNAs interact with miRNAs to exert regulatory control over the expression of the specific mRNA under consideration. Antisense lncRNAs can influence cancer development via this miRNA-mediated pathway (Rupaimoole and Slack, 2017). Prostate cancer (PCa) cells exhibit notable expression of the antisense long non-coding RNA (lncRNA) FOXP4-AS1 and its corresponding coding transcript FOXP4. Elevated levels of FOXP4-AS1 have been observed to promote cancer cell proliferation and are correlated with an unfavorable prognosis (Wu et al., 2019).

Interestingly, miR-3184-5p, which explicitly targets FOXP4, binds to FOXP4-AS1. The binding of miR-3184-5p by FOXP4 and FOXP4-AS1 is competitive. Therefore, it acts to increase FOXP4 protein levels. Numerous research studies have shown the tumor-promoting roles of ZEB1-AS1 (Lv et al., 2018); Two of them prove that ZEB1-AS1 is a ceRNA that promotes CRC cell growth and migration; however, the two targeted-miRNAs are distinct. A study revealed the presence of a negative correlation between the expression levels of miR-181a-5p in colorectal cancer (CRC) cells and the levels of ZEB1-AS1. Additionally, the study explored the role of the antisense lncRNA FOXC2-AS1 in doxorubicin resistance in osteosarcoma. Drug resistance in osteosarcoma cells was discovered to be facilitated by FOXC2-AS1, which functions as a ceRNA by binding competitively to miR-3607-3p and increasing FOXC2 expression (Zhang et al., 2017). Scientists looked at the breast cancer cell regulatory network for ceRNAs. They showed that SPRY4-IT1, an antisense lncRNA, acts as a ceRNA to enhance breast cancer cell proliferation by regulating ZNF703 expression via miR-211-3p sponging (Shi et al., 2015). Additional miRNA targets for ZEB1-AS1 are expected to be uncovered, given its involvement in carcinogenesis and progression in various cancer types. Furthermore, FOXC2-AS1 in PCa is a target of miR-1253. Furthermore, miR-324-5p uses TPT1-AS1 as a sponge in CC (Jiang et al., 2018). Future research in this field could lead to the creation of effective therapies, as there is a growing realization that several antisense long non-coding RNAs (LncRNAs) exert their functions in cancer through the competing endogenous RNA (ceRNA) pathway.

### 4.5 Genome imprinting

Genome imprinting is an epigenetic phenomenon that results in the differential expression of alleles depending on their parental origin. It plays a crucial role in mammalian development and has been implicated in various genetic disorders and diseases, making it an important area of study in genetics and developmental biology. Some genes are expressed monoallelically due to the epigenetic process known as genomic imprinting, in which only one of the two paternal alleles is active, and the other is repressed. This monoallelic expression pattern is set during gametogenesis and persists throughout the organism’s life (da Rocha and Gendrel, 2019). Genomic imprinting relies on regulating the expression of imprinted genes, and recent evidence suggests that antisense RNAs play a crucial part in this process (Manoharan et al., 2004). Imprinted genes frequently co-occur with natural antisense transcripts, with a frequency of 81% or more in one research (Katayama et al., 2005b). Over 160 imprinted genes have been discovered in both humans and mice, with the majority of them arranged in clusters. The process of directed chromatin and DNA alteration by antisense RNA spreads to include surrounding genes for several imprinted genes, including insulin-like growth factor type 2 receptor (igf2r) and the imprinting regulatory region of Kcnq1 (Sleutels et al., 2002; Pandey et al., 2008). The study investigated that Kcnq1ot1 is an imprinted noncoding RNA gene on mouse chromosome 7’s Kcnq1 domain, and its imprinting was studied to determine the function of antisense transcription in this process (Faghihi et al., 2010). Kcnq1ot1 is an essential regulator of the expression of several imprinted genes within the Kcnq1 domain and is expressed only from the paternal allele (Rosikiewicz and Makałowska, 2016). The lncRNA H19 was studied to see if it had a part in genomic imprinting in developing mice. The H19 gene locus has an ICR that controls transcription from the maternal allele. This gene is a well-known example of an imprinted gene. Specifically, they discovered that the father’s H19 ICR allele transcribes an antisense RNA. Proper H19 gene imprinting and preservation of the differential DNA methylation pattern at the H19 ICR rely on this antisense RNA (Kallen et al., 2013). Abnormal methylation patterns at the H19 ICR and changes in H19 gene expression were seen when the antisense RNA was deleted, demonstrating the critical involvement of antisense RNA in genomic imprinting at this locus (Schmitt and Chang, 2016). These effects are not caused by the RNAi pathway (Rossi and Paiardini, 2022). Similar to X chromosome inactivation (below), although with less penetrance, suppressive chromatin changes travel in both directions to nearby genes (Sleutels et al., 2002). Investigating the functions and regulatory mechanisms of antisense RNAs in genomic imprinting can deepen our understanding of gene regulation during development and disease. As the field progresses, we can expect discoveries and potential therapeutic implications from studying antisense RNA in genomic imprinting.

## 5 Applications of antisense RNA in anticancer treatments

Cancer is one of the leading causes of death in humans and one of the most rapidly spreading lethal diseases (Cavalli, 2013). Despite extensive research into cancer, early detection and treatment techniques are lacking (Xu J. et al., 2018). Cancer is widely recognized as a complex process that involves the inactivation of genes that suppress cancer and the activation of genes that promote cancer. Fortunately, evidence shows that antisense RNAs can be crucial in cancer detection and treatment (Hansen et al., 2015). Some ncRNAs may be helpful as diagnostic markers for cancer. Multiple studies have shown that aberrant expression of antisense RNA may be utilized as an indication in cancer detection, which is crucial for effective and clinically useful biomarkers in cancer treatment (Koch, 2014). One study demonstrated major differences in the expression levels of 15 miRNAs between healthy and malignant tissues. Finding new medications to control the expression of carcinogenic factors and associated genes is an urgent need for cancer patients awaiting effective therapies (Bustin and Murphy, 2013). It has been shown that Stat5 or survivin is overexpressed in several types of human cancer cells and primary tumors (Iorio et al., 2005). Therefore, several scientists use antisense RNAs to dampen Stat5 or survivin expression, reducing tumor cell proliferation and death (Wang T. H. et al., 2005). Some antisense RNAs have been shown to promote cancer (miR-21, miR-155, miR-17–20), while others have been found to decrease cancer (miR-15, miR-16, miR-143) (Zhang et al., 2012). Antisense RNA allows researchers to manipulate gene expression in two ways: they may increase the expression of tumor-suppressing genes or decrease the expression of cancer-promoting genes, respectively; this allows them to study the genes’ targets and determine the best ways to treat cancer. Antisense oligonucleotides (ASOs) are presently the most cutting-edge approach to therapeutic lncRNA targeting. These molecules are short stretches of DNA that may be rapidly constructed using criteria like sequence similarity and RNA accessibility (Lee and Mendell, 2020). ASOs have the ability to effectively trigger co-transcriptional cleavage mediated by RNase H at the binding site of the ASO. This process leads to premature termination of transcription and subsequently reduces the amounts of long non-coding RNA (lncRNA). While ASOs demonstrate high efficacy inside cellular environments, their clinical application is hindered by challenges such as *in vivo* toxicity and inadequate transport pathways, which restrict the effective targeting of therapeutic ASOs to tissues (Lai et al., 2020). Many ASOs undergo chemical modifications to enhance their hybridization affinity with target RNA, hence increasing resistance to nuclease degradation and reducing non-specific immunostimulatory activity. These modifications aim to improve the pharmacological properties of ASOs. GapmeR ASOs are one type of these chemical variants; they are triple-stranded RNA-DNA-RNA oligonucleotides in which some of the ribonucleotides have a 2′-O-methoxyethyl modified sugar backbone222 or other modifications such as locked nucleic acids and S-constrained ethyl residues (Seth et al., 2010).

### 5.1 Antisense oligonucleotide-based therapies

**FIGURE 4:**
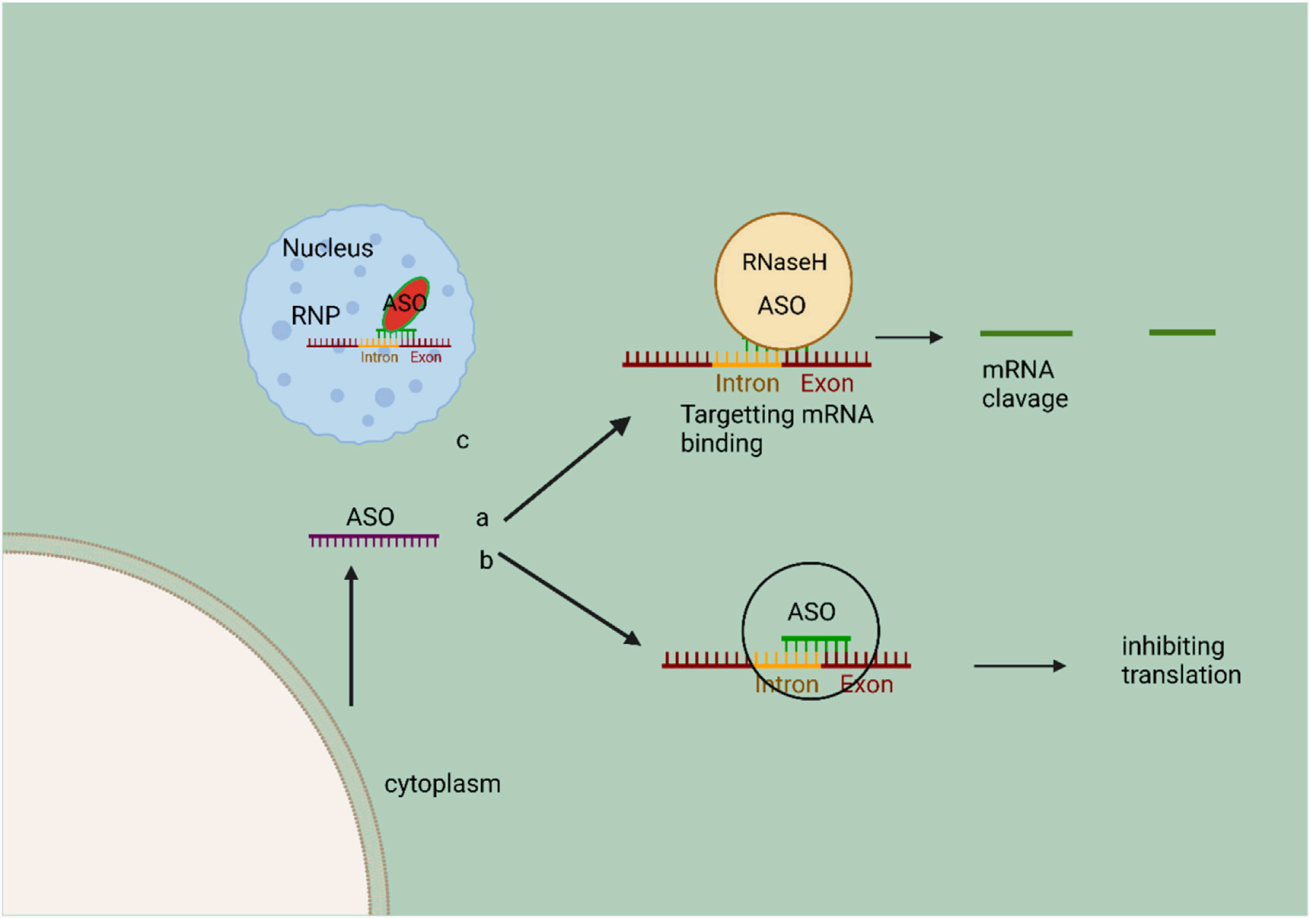
Schematic representation of the mechanism of action of ASO. In the cytoplasm, A, ASOs bind to specific RNA for RNase H cleavage, resulting in target mRNA destruction. In the cytoplasm, ASOs have the ability to bind to mRNA, thereby preventing RNA binding protein complexes from interacting and subsequently inhibiting the translation of target mRNA. ASOs, also known as antisense oligonucleotides, have the ability to penetrate the nucleus and regulate the process of splicing. This regulation occurs through the binding and obstruction of a splice junction or exonic/intronic sequences, resulting in the exclusion or inclusion of specific exons. The ribonucleoprotein (RNP) complex facilitates the catalysis of this process.

Synthetic antisense oligonucleotides (ASOs) are commonly used to trigger RNase H-mediated RNA degradation by forming a DNA-RNA hybrid with target RNA (Zhang et al., 2019). Occupancy-only ASOs may potentially trigger endogenous cellular monitoring programs that eliminate aberrant mRNAs, which we address in the application section (Ward et al., 2014). When ASOs operate on pre-mRNAs to generate mRNAs with premature termination codons (PTCs), the targets may be destroyed by nonsense-mediated mRNA decay (NMD) (Melton, 1985). In addition, ASOs may either halt or kick-start the translation process (Baker et al., 1999). ASOs, on the one hand (Liang et al., 2016), may silence the target RNA by altering polyadenylation or inhibiting translation at the 5′cap (Vickers et al., 2001). However, ASOs may also attach to inhibitory elements like uORF upstream of the target gene (Liang et al., 2017). Alternatively, other translation inhibitory elements (TIEs) might boost target RNA expression (Cereda et al., 2015). The pharmacological characteristics of ASOs may be enhanced using occupancy-only ASOs rather than those reliant on RNase H1 (Li and Rana, 2014). To avoid unwanted cleavage of target RNA by RNase H1 or Ago2, it is crucial that occupancy-only ASOs do not form an RNA-DNA duplex. One alternative commonly used in ASOs is phosphorodiamidate morpholino oligomers (PMOs), which replace the standard ribofuranose ring with a six-membered morpholino ring and have a backbone linked by phosphorodiamidates. This structural alteration safeguards the PMO against nucleases, while also minimizing the reduction in complementary RNA affinity (Crooke et al., 2021) (Figure 4). Furthermore, an additional design of (ASOs) is the RNase H independent or occupancy-only route. These ASOs are specifically engineered to act as steric hindrances, physically impeding or preventing the translation or splicing of the RNA molecule being targeted (Damase et al., 2021). The FDA and EMA have authorized a number of ASO medicines. Too far, nine ASO-based medications have been authorized for commercial usage, all of which treat uncommon disorders. However, the ASOs now under development demonstrate how the ASO platform is being used to treat other prevalent illnesses (Gagliardi and Ashizawa, 2021). Casimersen, the latest ASO-based medication to receive approval, was granted expedited clearance by the US Food and Drug Administration for the treatment of Duchenne muscular dystrophy (DMD). Frameshift or nonsense mutations in the DMD gene, which codes for the dystrophin protein, block functional dystrophin synthesis and cause Duchenne muscular dystrophy (DMD), a degenerative neuromuscular illness that is inherited via the X chromosome (Shirley, 2021). ASOs do not only focus on uncommon illnesses; they also aim to treat cardiovascular conditions. Researchers are also investigating ASO medication candidates for the treatment of cardiovascular disease (CVD), and they share certain similarities with siRNA therapeutics. Using RNase H1 dependent cleavage, Pelacarsen, a first-in-class GalNAc-conjugated PS ASO, specifically targets apolipoprotein(a) (apo(a)) mRNA (Wagner et al., 2021). The PMO medication candidate demonstrated good tolerability during a phase 1/2 study in individuals with DMD eligible for exon 45 skipping. All evaluable casimersen recipients showed an increase in exon 45 skipping, according to interim results from the phase 3 ESSENCE trial (NCT identifier: NCT02500381). However, the sample size is small, and the trial is still ongoing, making it difficult to determine whether or not Casimersen can improve motor function (Crooke et al., 2021). Elevated levels of lipoprotein(a), referred to as Lp(a), which is a lipoprotein similar to LDL composed of ApoB and apo(a) connected by a disulfide bridge, have been linked to an increased risk of cardiovascular disease. A study administered pelacarsen subcutaneously to 286 individuals with established cardiovascular disease (CVD) and elevated lipoprotein(a) [Lp(a)] levels. It was observed that Lp(a) levels decreased in a dose-dependent manner, ranging from 35% to 80%, with a favorable safety profile. Pelacarsen, an antisense oligonucleotide (ASO) medication candidate for CVD, has demonstrated promise in phase 2 trials and is currently undergoing evaluation in phase 3 studies (Fernandez-Prado et al., 2020). Recent developments in antisense technology have spurred the creation of ASOs, proving the adaptability and safety of ASO-based therapeutics. Many ASO candidates for treating more prevalent illnesses are undergoing cutting-edge clinical research and are expected to show promising results in the future. Unlike RNAi, which is ineffective against nuclear NATs, ASOs may effectively target nuclear NATs since RNase H is highly expressed there (Setten et al., 2019). In particular, the ASOs developed to block cis-NATs are known as antagonists (Derrien et al., 2012b). This may prevent sense transcripts from interacting with their antisense counterparts, leading to a breakdown of the NAT and transcriptional depression of the adjacent sense transcript (Derrien et al., 2012a). Loss-of-function studies on NATs have gained significant interest, but there are still significant barriers to their clinical applicability, including off-target effects, limited cellular uptake, and cytotoxicity (Arun et al., 2018). Fortunately, gene-editing technologies have advanced to the point where they may be used as an alternate method for NAT-targeted treatment.

### 5.2 Exploring the clinical implications and diagnostic/therapeutic opportunities of HOTAIR upregulation in cancer

At least 24 distinct forms of solid tumors have been associated with aberrant HOTAIR expression and function (Gupta et al., 2010). In cancer, increased HOTAIR expression is linked to worse outcomes in both overall and disease-free survival and metastasis and treatment resistance (Zhang et al., 2015). HOTAIR has been shown to support proliferation, survival, stemness, invasion, and resistance to treatment via interactions with molecules such as chromatin modifiers, ubiquitin ligases, and miRNAs (Sun et al., 2015). These shows that increased HOTAIR expression is a biomarker for cancer diagnosis, metastasis, therapeutic resistance, and poor survival (Heubach et al., 2015).

Given that HOTAIR is stable and detectable in bodily fluids and that its elevation has prognostic and diagnostic implications for cervical, breast, glioblastoma, pancreatic, and gastric cancers, as well as melanoma, it holds potential as a biomarker (Shen et al., 2018). Due to its ability to stimulate growth, survival, and invasion in several cancer cell types (Table 1), HOTAIR has emerged as a prospective therapeutic target (Marczynski et al., 2020). Accordingly, antisense oligonucleotides targeting HOTAIR have shown promise as a treatment in animal models. Breast, pancreatic, and gastric cancer cell growth and invasion may all be stopped using a small interfering RNA (siRNA) directed against HOTAIR (Kishikawa et al., 2015). Furthermore, it is enticing to consider selectively disrupting the interactions between HOTAIR and its partners in order to halt their carcinogenic activities. To make platinum more effective against ovarian tumors that have become resistant to chemotherapy, researchers developed a peptide-nucleic acid hybrid that blocks HOTAIR binding to EZH2 (Özeş et al., 2017). Therefore, expanding this approach to disrupt HOTAIR’s connections with additional partners in cancer cells is feasible. To fully harness HOTAIR’s diagnostic and therapeutic potential, it is essential to acquire a comprehensive understanding of how the expression and activity of HOTAIR become dysregulated in cancer cells. As a result, in the following paragraphs, we will examine why HOTAIR is malfunctioning with cancer.

### 5.3 CRISPR Cas9 and lncRNA in treatment of cancer

Tumors are caused by dysregulation of cell proliferation, including activating proto-oncogenes and inactivating tumor-suppressive genes (Skoulidis and Heymach, 2019). CRISPR/Cas9’s ability to alter numerous genes gives it significant potential in cancer treatment, and genome engineering technology offers fresh hope for cancer treatment (Martínez-Jiménez et al., 2020). With the advancements in CRISPR/Cas biology such as the discovery and characterization of diverse types of Cas proteins and gRNA engineering, the use CRISPR/Cas technology to knockout oncogenes have become a very effective way to prevent tumor growth. For instance, fixing tumor suppressor genes and restoring their function can be used to prevent tumor formation (Azangou-Khyavy et al., 2020). Lung, colorectal, head, and hepatocellular carcinoma research currently focuses on CRISPR/Cas9-based gene therapy (Lu Y. et al., 2020; Xu et al., 2020). Recent research has indicated that the utilization of CRISPR/Cas9 has demonstrated efficacy in effectively suppressing the transcriptional activity of loci that express long non-coding RNAs (lncRNAs) (Koch, 2017). CRISPR/Cas9 system has been employed to specifically target the gene promoters in order to induce transcriptional silencing. Research has indicated that a substantial number of over 16,000 lncRNA promoters throughout the human genome have the potential to be selectively influenced by guide RNAs (Gilbert et al., 2014). In addition to target the promoters, CRISPR/Cas9 system has been employed for specifically targeting genomic DNA within cancer cells and animal cancer models. For instance, the metastatic process was significantly impeded upon the deletion of lncRNA-NEAT1 and lncRNA-MALAT1 (Thakore et al., 2015). Previous studies have revealed that lncRNA-GMAN, a long non-coding RNA, is associated with gastric cancer metastasis. The GMAN gene exhibits notably increased expression levels within gastric cancer cells. This heightened expression of GMAN has been found to be correlated with an unfavorable prognosis (Liu et al., 2017). A meticulously designed proof-of-concept animal experiment highlighted the effectiveness of a GMAN-targeting CRISPR/Cas9 system in inhibiting the spread of gastric cancer cells and improving the overall survival rate in murine subjects (Adriaens et al., 2016).

The CRISPR/Cas9 system has extensive flexibility and precise targeting capabilities as a genome-editing tool in theory. However, it is important to note that in real implementations, there is still a possibility of off-target cleavage events occurring. Hence, it is imperative for oncologists to exercise increased prudence while formulating gene-editing treatment. The clinical use of the CRISPR/Cas9 system for targeting lncRNA in cancer treatment may lack clarity. In addition, it is crucial to prioritize the development of gene-editing technologies that are more precise and tailored to specific genetic modifications.

### 5.4 RNAi-based therapy

Too far, the Food and Drug Administration (FDA) has granted authorization for three small interfering RNA (siRNA) drugs, namely, patisiran, givosiran, and lumasiran. Additionally, there are currently seven siRNA candidates, including inclisiran, vutrisiran, fitusiran, cosdosiran, nedosiran, tivanisiran, and teprasiran, that are undergoing Phase III clinical research. Patisiran is a significant milestone as the inaugural RNAi-based medication sanctioned by the FDA for the management of hereditary transthyretin-mediated polyneuropathy. This achievement heralds a promising era for the development and application of RNAi therapies (Mitsuyasu et al., 2009; Adams et al., 2018). The patisiran small interfering RNA (siRNA) molecule, also known as ALN-18328, functions in a manner akin to inotersen by effectively suppressing any potential messenger RNAs (mRNAs) that exhibit coding region modifications. This is achieved through the specific targeting of the (3UTR) of the transthyretin (TTR) gene (Benson et al., 2018). Alnylam developed the stabilization chemistry-GalNAc delivery platform with the objective of augmenting the therapeutic efficacy of siRNA therapeutics (Adams et al., 2018). As of the present time, over 33% of RNA interference (RNAi) medications undergoing clinical trials consist of small interfering RNAs (siRNAs) coupled with GalNAc (Zimmermann et al., 2017). Revusiran, a GalNAc-siRNA medication, shown a notable enhancement in the absorption of asialoglycoprotein receptors for hepatic administration, marking it as the pioneering medicine in this category (Garber, 2016). Nevertheless, the “ENDEAVOUR” phase III clinical research (NCT02319005) was terminated due to an inequitable dispersion of mortality rates (Nair et al., 2017). Alnylam has continued to create GalNAc-siRNA conjugates for therapeutic use despite the failure of revusiran by carefully integrating chemical changes into the siRNA that can provide further stability against nuclease activity (Markham, 2021). Givosiran and Lumasiran, the second and third small interfering RNA (siRNA) medications that have received approval from the Food and Drug Administration (FDA), have provided evidence indicating that GalNAc-conjugated siRNAs given subcutaneously are associated with favorable tolerability, substantial reduction in target mRNA levels, and a low risk profile (Morrison, 2018).

Moreover, numerous well-established corporations have implemented sophisticated RNA interference (RNAi) techniques, involving comprehensive chemical modifications and metabolic stabilization. These techniques incorporate various secondary structures and patterns of chemical alteration. RNA interference (RNAi) therapy has demonstrated significant potential in treating a broad spectrum of disorders, extending beyond liver-related conditions. It can effectively target both rare and common diseases that afflict a substantial number of patients. One exemplar corporation proficient in the development of pharmaceuticals is Quark Pharmaceuticals. They have successfully formulated specialized medications to combat kidney damage (referred to as QPI-1002) and various eye ailments (known as QPI-1007) (Khvalevsky et al., 2013; Mathison et al., 2017). Presently, there has been a rapid change in the pharmaceutical industry’s attention towards the development of RNA interference (RNAi) medications for treating cancer. The SiG12D LODER (Local medication EluteR) is a biodegradable polymeric matrix that incorporates the KRASG12D siRNA (siG12D) medication. The purpose of this medication is to intervene therapeutically in pancreatic ductal adenocarcinoma, as evidenced by clinical trial identifier NCT01188785. Furthermore, the development of TKM-080301, a Plk1 inhibitor, had been undertaken for the treatment of hepatocellular carcinoma (Titze-de-Almeida et al., 2017). Furthermore, Atu027, designed to target protein kinase N3, has been explored for its potential effectiveness against advanced solid tumors. Well beyond siRNA. MicroRNA inhibitors (also known as Anti-miRs) and microRNA mimics have the potential to modulate the expression of microRNAs, by either suppressing or enhancing their activity (Samir and Pessler, 2016). Miravirsen (SPC3649) and RG-101 are antimiRs designed to target miR-122 in order to cure infections caused by the hepatitis C virus. MRX34, also known as the miR-34a mimic, represents a significant milestone as the inaugural miRNA medicine specifically designed to target cancer (Drury et al., 2017). However, it is crucial to acknowledge that none of these methods is currently employed in clinical practice. In conclusion, the incorporation of RNA chemical modifications and the utilization of nanocarrier systems hold promise in improving the effectiveness of RNA drug delivery. Further investigation into RNA-based therapies, encompassing the utilization of RNA molecules as therapeutic agents and the precise targeting of RNA using small molecules, has the potential to broaden the range of RNA-based therapeutics accessible for patient treatment.

### 5.5 saRNA therapeutics

**FIGURE 5:**
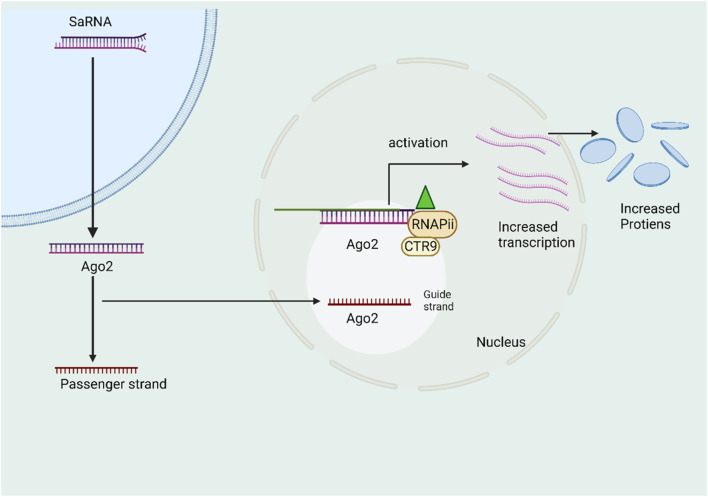
The schematic picture illustrates the mechanism of action of self-amplifying RNA (saRNA). The saRNA molecule is introduced and subsequently loaded into the Ago2 protein. This process is then followed by the selection of a certain strand of the saRNA molecule. The Ago2 protein cleaves the selected strand, resulting in the formation of the saRNA/Ago2 complex. Finally, this complex is translocated into the nucleus. The RITA complex, in its active state, selectively interacts with and forms a complex with the promoter region of the genomic target locus. This complex also interacts with RHA and CTR9, resulting in the activation of transcriptional activity through the involvement of RNA Polymerase II (RNAP II).

The category of diminutive double-stranded RNA (dsRNA) encompasses a subgroup known as small activating RNA (saRNA), which was initially documented by (Li et al., 2006). The researchers made a discovery that involved the use of 21-nt dsRNAs to target certain gene promoters, resulting in the induction of transcriptional gene activation. This phenomenon was subsequently termed RNA activation (RNAa). Both findings elucidated a naturally occurring phenomenon involving the activation of gene expression through the mediation of tiny duplex RNA (Janowski et al., 2007). The proposed 21-nucleotide double-stranded RNA (dsRNA) that is complementary to the promoter region of E-cadherin, p21, and VEGF genes has been observed to increase gene expression in a manner that is specific to the sequence. This induction is dependent on Ago2, similar to the mechanism of RNA interference (RNAi) (Li et al., 2006). The study exhibited an observed increase in the expression of the progesterone receptor with the use of complementary duplex RNAs that specifically targeted the promoter region of the receptor (Janowski et al., 2007; Huang et al., 2010). Furthermore, it has been demonstrated that RNAs is conserved throughout a diverse range of mammalian cell types. There exist molecular parallels between the mechanisms of RNA interference (RNAi) and RNA activation (RNAa), as observed. In the context of RNAa, small activating RNAs (saRNAs) are incorporated into the Ago2 protein, resulting in the formation of the RNA-induced transcriptional activation (RITA) complex (Portnoy et al., 2016). The saRNA/Ago2 complex, RNA helicase A, and the RNA polymerase-associated protein CTR9, which interacts with RNA Pol II to promote transcriptional initiation and productive elongation, make up the RITA complex (Portnoy et al., 2016). RNAa exhibits unique characteristics in terms of its molecular dynamics, its ability to target specific regions of the genome, and its capacity to activate the elongation of target gene transcription within the nucleus (Ghanbarian et al., 2021). The identification of RNAa and the involvement of small activating RNA (saRNA) provide novel perspectives in the investigation of targeted gene activation. Moreover, self-amplifying RNA (saRNA) presents itself as an innovative therapeutic approach for increasing gene expression in disorders characterized by suppressed transcriptional activity (Voutila et al., 2017). While the majority of candidates remain in the preclinical phase, the compound MTL-CEBPA, which has been developed by MiNA Therapeutics, has progressed to phase2. This advancement involves the utilization of sorafenib, a protein kinase inhibitor, in combination with MTL-CEBPA for treating patients who are afflicted with advanced (HCC) due to infection from hepatitis B or C. The NCT identifier (NCT04710641) identifies this clinical trial. MTL-CEBPA is a novel synthetic RNA molecule known as a saRNA oligonucleotide. It possesses a 2′-O-Me alteration and is encased within liposomal nanoparticles called SMARTICLES. This therapeutic agent is provided to patients with (HCC) via intravenous infusion (Sarker et al., 2020). The transcription element C/EBP-a is a leucine zipper protein that primes and activates myeloid gene expression by binding to the promoter region of the myeloid gene, which is known to be involved in hepatocyte regulation. Researchers discovered C/EBP-a downregulation in myeloid-derived suppressor cells in murine tumor models, while C/EBP-a upregulation reduced tumor growth in liver cancer animal models (Reebye et al., 2018). To ascertain supplementary protein contents linked to saRNA effector complexes, the researchers utilized a ChIP-based biotinylated RNA pull-down experiment (ChIbRP) in combination with mass spectrometry (MS) (Portnoy et al., 2016). Within the group of 42 proteins, there are two distinct proteins, CTR9 (RNA polymerase-associated protein CTR9 homolog) and RHA (nuclear DNA helicase II), that have been verified as transcriptional activators. These proteins exhibit the capacity to unravel and attach to DNA/RNA molecules. These proteins have been validated as interactors with RNAP II. In the context of immunoprecipitation tests, it was observed that two proteins located in the nucleus were co-immunoprecipitated with both AGO2 and RNAP II when saRNAs were used as therapy. This finding suggests a robust link between the saRNA-AGO2 complex and the proteins RHA, CTR9, and RNAP II. In brief, AGO2 facilitates the loading of the guide strand of saRNAs, resulting in the formation of a functional saRNA-AGO2 complex (Portnoy et al., 2016). The binding of saRNA-loaded AGO2 to the promoter region leads to the induction of an open chromatin structure and the recruitment of the CTR9 and RHA complex. The process of RNA-induced transcriptional activation (RITA), facilitated by AGO2, initiates transcription by inducing phosphorylation of RNA polymerase II at Ser2 and ubiquitination of histone H2B (Yoon and Rossi, 2018). At present, the prevailing conceptual framework on the molecular mechanisms underlying the activation of gene expression by synthetic activating RNA (saRNA) is illustrated. If we enhance the targeting area of the gene may focus on improving the precision and specificity of saRNA targeting, minimizing off-target effects (Figure 5).

## 6 Discussion and future prospective

In a specific context, asRNAs are a group of naturally occurring single-stranded transcripts. They have the ability to attach to their corresponding sense RNA (mRNA) through sequence complementarity, thus effectively inhibiting mRNA translation and protein synthesis. It is worth noting that asRNAs can be considered the same as natural antisense transcripts (NATs). However, in a more general context, asRNAs encompass a wider array of RNA types. These RNA types regulate the abundance and functions of their target RNAs through sequence complementarity. As a result, they serve as a shared mechanism that ultimately modifies gene expression. This broader perspective suggests that NATs, mRNAs, miRNAs, siRNAs, and competing endogenous RNAs (ceRNAs) all have the potential to function as antisense RNAs (Wang et al., 2019). asRNAs have consistently shown rapid growth and are recognized as significant regulators of gene expression within complex regulatory networks. The dysregulation of NATs has been observed in numerous malignancies and has been associated with a wide range of malignant phenotypes (Modarresi et al., 2012). Recent studies have highlighted the growing significance of NATs in the fundamental characteristics of cancer. These NATs have been found to exert their influence by modulating post-transcriptional modifications (PTMs) of messenger RNAs (mRNAs) and regulating epigenetic alterations. Notably, numerous preclinical investigations have indicated the promising potential of various strategies such as RNAi, artificial splicing, and CRISPR-based techniques for targeting NATs (Liu et al., 2018). Evidence supports the existence of both oncogenic and tumor-suppressive asRNAs. With the advancement of our understanding of asRNAs, new opportunities have emerged for leveraging asRNA technology in the research and development of innovative approaches for cancer treatment.

However, given the extensive pool of antisense RNAs (asRNAs) consisting of numerous diverse classes, our current knowledge of asRNAs barely scratches the surface. Therefore, there is a pressing need to deepen our understanding of asRNAs in order to effectively utilize them for future anticancer therapies. Subsequent research will focus on the following factors:

The discovery and confirmation of new asRNAs will expand the already substantial collection of established asRNAs. Particularly, the groundbreaking concept that messenger RNAs can function as asRNAs necessitates a reevaluation of our assumptions regarding the role of RNA and prompts the search for mRNAs with asRNA capabilities.

To enhance our comprehension of asRNA function in carcinogenesis, it is crucial to conduct tissue- and cell-specific expression profiling of asRNAs in various malignancies and at different stages of cancer.

To attain a comprehensive understanding of asRNA functions in cancer biology, as well as their mechanisms of action, it is essential to broaden our investigations to encompass both existing and yet-to-be-discovered asRNAs, conducting further validation and characterization.
